# Effects of Inhibition of Interleukin-6 Signalling on Insulin Sensitivity and Lipoprotein (A) Levels in Human Subjects with Rheumatoid Diseases

**DOI:** 10.1371/journal.pone.0014328

**Published:** 2010-12-13

**Authors:** Olaf Schultz, Frank Oberhauser, Jasemine Saech, Andrea Rubbert-Roth, Moritz Hahn, Wilhelm Krone, Matthias Laudes

**Affiliations:** 1 Department of Internal Medicine II and Centre of Molecular Medicine Cologne, University of Cologne, Cologne, Germany; 2 Department of Internal Medicine I, University of Cologne, Cologne, Germany; 3 Institute of Medical Statistics, Informatics and Epidemiology, University of Cologne, Cologne, Germany; 4 CECAD-Cluster of Excellence in Cellular Stress Responses in Aging Associated Diseases, University of Cologne, Cologne, Germany; University of Bremen, Germany

## Abstract

**Background:**

Interleukin-6 (IL-6) is a pro-inflammatory cytokine that has been found to be increased in type 2 diabetic subjects. However, it still remains unclear if these elevated IL-6 levels are co-incidental or if this cytokine is causally related to the development of insulin resistance and type 2 diabetes in humans. Therefore, in the present study we examined insulin sensitivity, serum adipokine levels and lipid parameters in human subjects before and after treatment with the IL-6 receptor antibody Tocilizumab.

**Methodology/Principal Findings:**

11 non-diabetic patients with rheumatoid disease were included in the study. HOMA-IR was calculated and serum levels for leptin, adiponectin, triglycerides, LDL-cholesterol, HDL-cholesterol and lipoprotein (a) (Lp (a)) were measured before as well as one and three months after Tocilizumab treatment. The HOMA index for insulin resistance decreased significantly. While leptin concentrations were not altered by inhibition of IL-6 signalling, adiponectin concentrations significantly increased. Thus the leptin to adiponectin ratio, a novel marker for insulin resistance, exhibited a significant decrease. Serum triglycerides, LDL-cholesterol and HDL-cholesterol tended to be increased whereas Lp (a) levels significantly decreased.

**Conclusions/Significance:**

Inhibition of IL-6 signalling improves insulin sensitivity in humans with immunological disease suggesting that elevated IL-6 levels in type 2 diabetic subjects might be causally involved in the pathogenesis of insulin resistance. Furthermore, our data indicate that inhibition of IL-6 signalling decreases Lp (a) serum levels, which might reduce the cardiovascular risk of human subjects.

## Introduction

The prevalence of obesity and type 2 diabetes is increasing rapidly in most industrialised countries throughout the world [1]. These metabolic abnormalities can lead to alterations within the vascular wall inducing atherosclerosis [2] which may result in myocardial infarction, stroke and renal dysfunction thereby reducing life expectancy [3]. An increasing number of clinical and experimental studies suggest that inflammatory mechanisms are important in the pathogenesis of type 2 diabetes. For example, it has been shown that in obese and type 2 diabetic human subjects adipocytes secrete increasing levels of chemokines compared to lean controls [4]. Of these, *monocyte chemotactic protein* (MCP)-1 is known to attract blood monocytes into adipose tissue where they transform into macrophages [5]. These cells are able to secret a huge array of pro-inflammatory cytokines, e. g. *tumor necrosis factor* (TNF)-α and *interleukins* (IL) [5]. Of these, TNF-α has been shown to alter adipogenesis [6] and to inhibit insulin signalling in mice [7] suggesting that low-grade inflammation induces insulin resistance.

Within the last decade, monoclonal antibodies against specific pro-inflammatory cytokines have become increasingly important in the treatment of severely active immunological diseases. For example, the anti-TNF-α antibodies infliximab [8], adalimumab [9], golimumab [10] and certolizumab [11] are commonly used in clinical practice for the treatment of patients with rheumatoid arthritis. Besides these monoclonal antibodies, other so-called “biologicals” have been developed, e. g. the soluble TNF-α receptor etanercept [12] as well as the recombinant IL-1 receptor antagonist anakinra [13], which is known to be highly effective in cases of Still's syndrome.

Biologicals are not only very effective drugs for treatment of immunological diseases but are also very elegant tools for translational research. Since the cytokines causing classical autoimmune diseases like rheumatoid arthritis are also thought to promote low-grade inflammation in the pathogenesis of insulin resistance and type 2 diabetes, clinical investigation of the effects of TNF-α and interleukin-1 inhibitors in human subjects on metabolic parameters has previously been undertaken. For example, Rosenvinge A. et al. examined insulin sensitivity by hyperinsulinaemic euglycaemic clamp studies in patients with rheumatoid arthritis before and 8 weeks after adalimumab treatment [14]. Interestingly, they did not observe a significant effect of anti-TNF-α treatment on insulin sensitivity. This finding has been confirmed by Dominguez et al. in a cohort of 20 obese patients with type 2 diabetes where etanercept treatment did not affect insulin sensitivity [15]. These observations raise the question, whether low-grade inflammation is important at all in the development of type 2 diabetes in the human organism or if elevated inflammatory markers are only epiphenomena. However, in a double-blinded, parallel-group trial involving 70 patients with type 2 diabetes anakinra treatment for 13 weeks lowered glycosylated haemoglobin-A1c levels by 0.46% compared to placebo [16]. Interestingly, this improvement of glycemic control was not due to enhanced insulin sensitivity but rather due to improved β-cell secretory function [16] suggesting that IL-1 is more involved in maintaining β-cell homeostasis than in adipose tissue biology in human subjects with type 2 diabetes.

Of the many interleukins identified, IL-6 exhibits a strong association with insulin resistance in human subjects [17]. In 2009 a monoclonal antibody against the IL-6 receptor, called Tocilizumab, was approved for treatment of rheumatoid arthritis in Europe. Since translational research within the last few years failed to demonstrate an important role for TNF-α in inducing insulin resistance in humans, in the present study we aimed to examine if IL-6 might be of clinical relevance in this context by measuring insulin sensitivity, adipokine serum levels and lipid parameters in patients with immunological disease before and at 1 and 3 months of Tocilizumab therapy.

## Methods

### Study cohort

The study was approved by the local ethics committee (Ethik Kommission, Universität zu Köln). Written informed consent was obtained from each subject before including into the study. A total number of n = 11 human subjects with rheumatoid disease were included. 7 were females, 4 were men. Mean age was 50.7±4.4 years. Inclusion criteria were: age between 20 and 70 years and caucasian descent. Exclusion criteria were: acute or chronic infections, cancer, elevations of liver indices more than 3-fold greater than the top of the normal range, serum-creatinine levels >1.5 mg/dl, or pregnancy. Mean body mass index (BMI) was 28.0±1.8 kg/m^2^. None of the patients was diagnosed with type 1 or type 2 diabetes. All patients were treated with steroids at doses of ≤10 mg/d prednisone equivalent (table 1). Patients were advised not to change the steroid dosage throughout the 3 month study period.

**Table 1:** Demographic, anthropometric and clinical characteristics of the study population.

patient	sex	age (a)	weight (kg)	hight (m)	BMI (kg/m2)	body fat (%)	current medication
1	f	67,00	54,00	1,54	22,77	36,60	Prednisone 5 mg/d, Methotrexate 10 mg/week
2	m	38,00	113,00	1,88	31,97	34,90	Prednisone 7,5 mg/d, Colchicin 1,5 mg/d, Allopurinol 300 mg/d, Pantoprazol 20 mg/d
3	m	64,00	73,00	1,82	22,04	20,50	Prednsione 10 mg/d, Leflunomide 20 mg/d, Omeprozol 20 mg/d, Simvastatin 40 mg/d
4	m	60,00	119,00	1,75	38,86	41,10	Prednisone 10 mg/d, Simvastatin 40 mg/d, Enalapril 5 mg/d, Torasemide 10 mg/d
5	f	55,00	92,00	1,68	32,60	50,00	Prednsione 10 mg/d, Leflunomide 20 mg/d
6	f	44,00	61,00	1,73	20,38	34,50	Methyl-Prednisolone 4 mg/d
7	m	41,00	94,00	1,83	28,07	32,90	Prednisone 5 mg/d, Leflunomide 10 mg/d, Esomeprazol 20 mg/d
8	f	69,00	81,00	1,64	30,12	-	Prednsione 10 mg/d, Alendronate 70 mg/week
9	f	48,00	64,00	1,69	22,41	39,10	Prednisone 5 mg/d
10	f	21,00	78,00	1,76	25,18	44,40	Prednisone 10 mg/d, Leflunomide 20 mg/d, Omeprazol 20 mg/d, Phenprocoumone 1,5 mg/d
11	f	44,00	90,00	1,68	31,89	48,50	Prednsione 5 mg/d

BMI  =  body mass index, f =  female, m =  male. Means ± SEM.

### Biochemical analysis

Blood samples were taken after a 12 h fasting period, centrifuged at 1000 rpm and serum samples were stored at −80°C before measurements were performed. Glucose levels were measured by the oxidase method. Triglycerides were determined by enzymatic colorimetric assay (Roche Diagnostics). LDL- and HDL-cholesterol were measured by a homogeneous direct method (Roche Diagnostics). Lipoprotein (a) (Lp (a)) was determined by turbidometry (Roche Diagnostics). C-reactive Protein levels were also measured by turbidometry (CRPL3, Roche Diagnostics) with an analytical sensitivity of 0.3 mg/l. Insulin and IL-6 levels were measured by chemiluminescence (Immulite 2000 insulin, Siemens (Germany), analytical-sensitivity: 2 µIU/ml, Immulite IL-6, Siemens (Germany), analytical-sensitivity: 2 pg/ml). ELISA for adipokine measurement were purchased from IBL International as follows: Adiponectin ELISA order number: BV51001 (analytical-sensitivity: 0,2 µg/ml), Leptin ELISA order number RE53151 (analytical-sensitivity: 1,0 ng/ml). The ELISA were performed according to the instructions of the manufacturer.

### Body composition analysis

Body fat content was analyzed by dual-X-ray-absorptiometry (Lunar-Prodigy-Advance (GE Healthcare)).

### Statistical analysis

The groups were compared using Wilxocon-Tests. Correlation was examined using Spearman's rank correlation coefficient. p<0.05 was considered to be significant. Data are given as mean ± SEM. The HOMA-IR was calculated as follows: fasting insulin (µU/ml)*fasting glucose (mg/dl)/405.

## Results

### Effect of IL-6 inhibition on inflammatory parameters in human subjects

**Figure 1:**
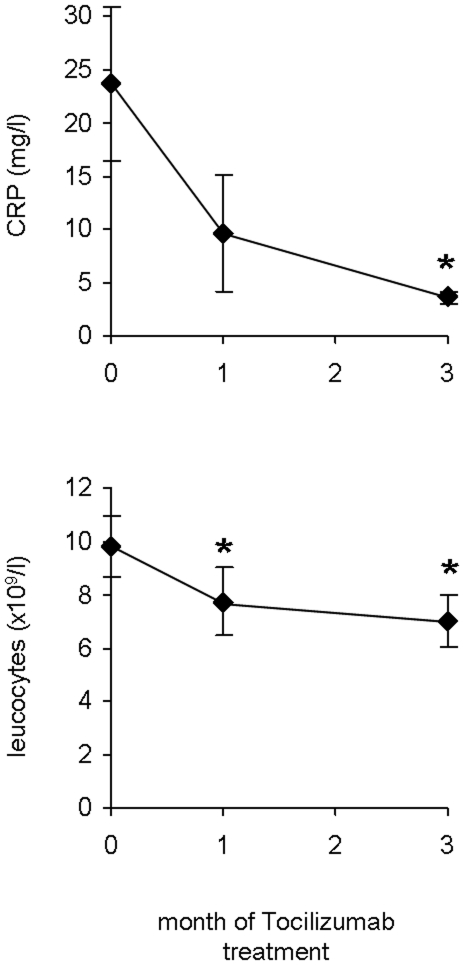
Effect of IL-6 inhibition on inflammatory parameters in human subjects. Data are expressed as Means ± SEM. * = p<0.05.

Tocilizumab is a monoclonal antibody against the IL-6 receptor which is given at a dosage of 8 mg/kg body weight intravenously for a 4 weeks period. In order to ensure reliable effects of this antibody in the subjects examined, we first measured IL-6 serum levels during Tocilizumab treatment. At baseline, IL-6 levels were measured as 18.0±8.5 pg/ml with a minimum of 2.0 pg/ml and a maximum of 98.0 pg/ml. As expected, blockade of the IL-6 receptor resulted in compensatory up-regulation of IL-6 serum levels to 34.5±8.5 pg/ml after one month and 39.2±13.2 pg/ml after three months of therapy which has been shown in Tocilizumab treated patients before [18]. We next compared C-reactive protein levels and leukocyte cell count before and after three months of treatment. As shown in figure 1, both inflammatory parameters decreased significantly due to Tocilizumab therapy. In respect to the clinical response 8/11 patients reported a significant reduction in symptoms of their rheumatoid disease. In these patients, Tocilizumab therapy was prolonged for more than 6 months after the end of the present study. 3/11 patients reported no or only mild improvement of clinical symptoms and Tocilizumab therapy was discontinued after the three months of the study period.

### Effect of IL-6 inhibition on body mass index and adipokine serum levels in human subjects

**Figure 2:**
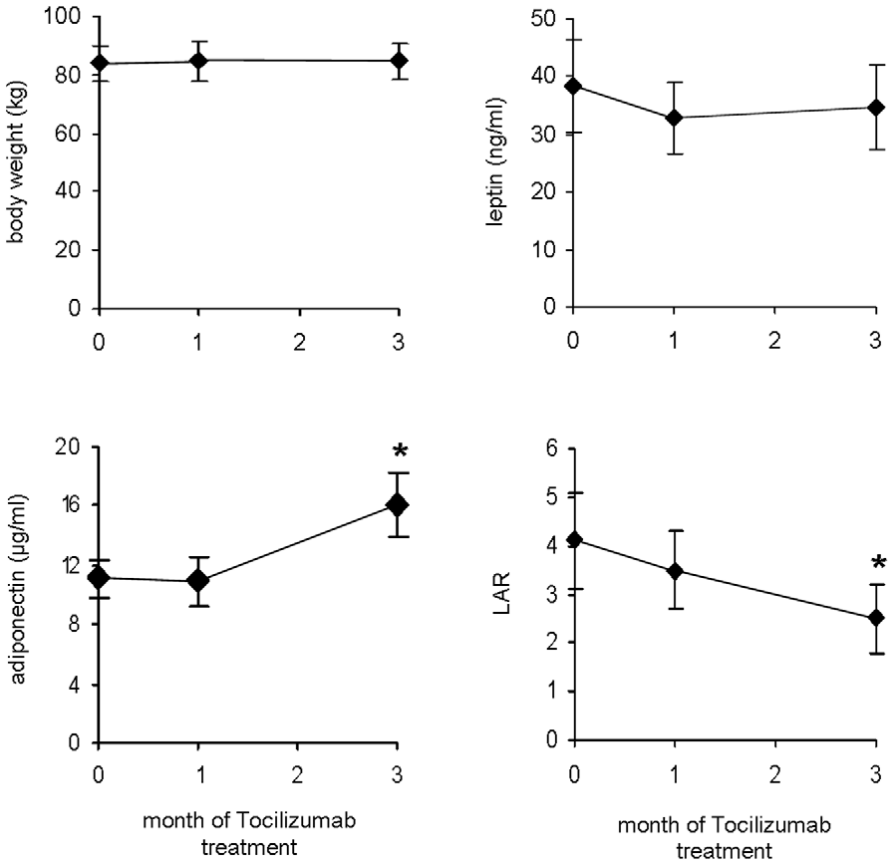
Effect of IL-6 inhibition on body weight and adipokine serum levels in human subjects. Data are expressed as Means ± SEM. * = p<0.05.

Having shown a significant immunological response of the subjects to Tocilizumab, we next aimed to examine metabolic parameters. Mean body mass index was 28.0±1.8 kg/m^2^ before and 28.3±1.7 kg/m^2^ after 3 months of treatment and therefore was not significantly altered due to inhibition of IL-6 signalling. Leptin levels at baseline were 38.35±8.0 ng/ml and were not significantly altered throughout the study period (fig. 2) which is in agreement with previous reports showing that leptin levels are closely related to body mass index [19]. Adiponectin levels were unchanged within the first month of treatment, but exhibited a significant up-regulation after 3 months of Tocilizumab therapy (fig. 2). The ratio of leptin to adiponectin (LAR) therefore accounted to 4.1±1.0 at baseline and was reduced to 3.5±0.7 after one month and 2.6±0.7 after three months of the study period.

### Effect of IL-6 inhibition on insulin sensitivity of human subjects

**Figure 3:**
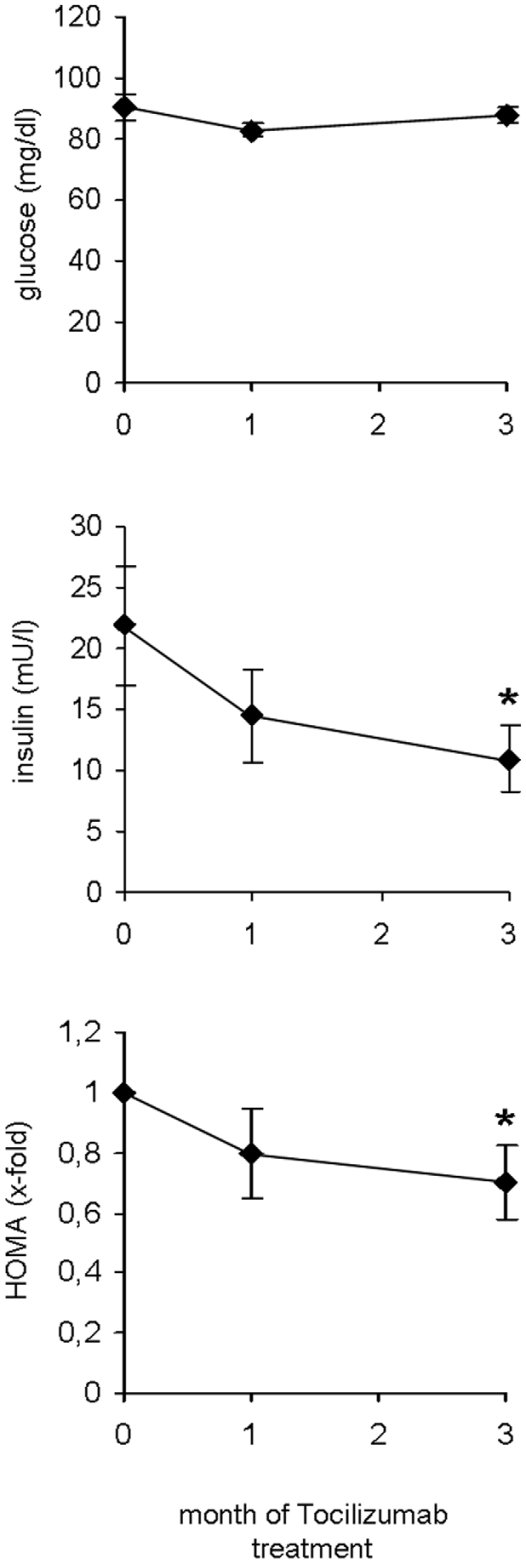
Effect of IL-6 inhibition on insulin sensitivity of human subject. Data are expressed as Means ± SEM. * = p<0.05.

We next aimed to investigate if insulin sensitivity is affected by inhibition of IL-6 signalling in human subjects with rheumatoid diseases. Therefore we first measured fasting plasma glucose and fasting serum insulin levels and calculated the HOMA-IR (fig. 3). When the study was started, mean HOMA-IR was 4.9±1.1 which reflects a moderate degree of insulin resistance of the study population, which is most likely due to inflammation and/or glucocorticoid treatment. Of importance, the patients were advised not to change the steroid dosage throughout the study period to ensure the metabolic effects observed are solely due to Tocilizumab. After the first month of treatment the HOMA-IR did not significantly change compared to baseline (fig. 3). However, at the end of the study period the HOMA-IR significantly decreased, indicating enhanced insulin sensitivity due to inhibition of IL-6 signalling. To substantiate this finding we also calculated the LAR, a novel marker for insulin resistance [20]. In complete agreement with the HOMA-IR, the LAR was unaltered within the first month of the study period but was significantly reduced after 3 months of Tocilizumab therapy (fig. 2). Mean glycosylated HbA1c levels of these non-diabetic patients were within the normal range at baseline and did not change significantly throughout the study period (table 2). Taken together, the two independent parameters, HOMA-IR and LAR, both suggest that inhibition of IL-6 signalling in human subjects with rheumatoid diseases improves insulin sensitivity.

**Table 2:** Metabolic parameters of the study population.

	0 month	1 month	3 month
TG (mg/dl)	141.9±15.1	170.8±26.2	169.6±27.0
LDL (mg/dl)	122.5±11.2	122.7±10.7	133.1±12.0
HDL (mg/dl)	53.6±6.5	61.0±5.0	63.0±5.1
Lp(a) (mg/dl)	34.5±12.8	24.3±7.6(a)	19.9±6.3(b)
Glucose (mg/dl)	90.5±4.5	82.7±2.1	87.5±2.7
HbA1c (%)	5.4±1.5	5.3±0.1	5.2±0.1

TG =  triglycerides, LDL =  LDL-cholesterol, HDL =  HDL-cholesterol, HbA1c =  glycosylated haemoglobin A1c levels.

(a) =  p<0.05 compared to 0 month,

(b) =  p<0.05 compared to 0 month. Means ± SEM.

### Effect of IL-6 inhibition on lipid parameters in human subjects

**Figure 4:**
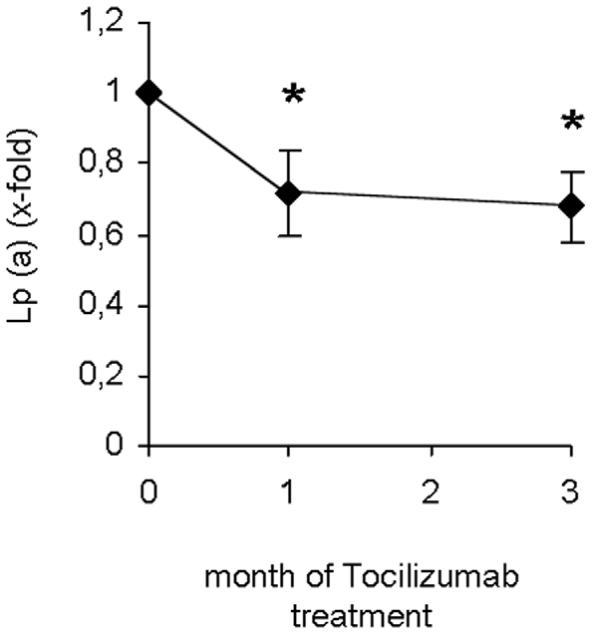
Effect of IL-6 inhibition on lipoprotein (a) levels in human subjects. Data are expressed as Means ± SEM of lipoprotein (a) levels x-fold compared to time point 0 month. * = p<0.05.

In previous reports it has been suggested that cholesterol levels might be altered by Tocilizumab treatment [21]. In this respect we also noticed a slight increase in LDL as well as HDL cholesterol levels (table 2). Also, triglyceride levels were found to be slightly elevated during treatment (table 2). However, these changes did not reach statistical significance. In addition, we measured Lipoprotein (Lp) (a) levels in the present study since this metabolite is known to be an independent cardiovascular risk factor. Mean Lp (a) levels were 34.5±12.8 mg/dl when the study was started. Of importance, already after one month of treatment, Lp (a) levels decreased significantly. As shown in figure 4, this effect persisted till the end of the study suggesting that IL-6 signalling is involved in the regulation of Lp (a) metabolism in humans.

## Discussion

Since 2001 several population based studies have shown a positive correlation of IL-6 serum levels and insulin resistance in human subjects [17], [22]–[24]. However, the association of IL-6 and type 2 diabetes is not well understood to date, as several studies in rodents and humans have reported contradictory results. For example, a common polymorphism in the IL-6 gene, -174G>C, has been found to be associated with risk for type 2 diabetes in the majority of population-based human studies [25]. However, in the largest of these studies performed in Denmark in 2005, an independent association of the *IL6-174G* allele with type 2 diabetes was not found [26]. Furthermore, targeted ablation of the IL-6 gene in one mouse model resulted in mature onset obesity and insulin resistance [27] while a second, independent study on IL-6^−/−^ mice, did not exhibit differences in body weight compared to wild type animals [28]. In addition to genetic modifications of rodents, studies have been reported using neutralizing IL-6 antibodies in mice, which is an experimental approach comparable to our study design. Interestingly, in these experiments, inhibition of IL-6 signalling in obese mice increased hepatic insulin sensitivity [29], suggesting that IL-6 in an obese organism might cause insulin resistance.

IL-6 is a 26 kD protein that acts through a receptor complex consisting of two subunits, gp130 and a ligand specific alpha chain, IL-6Rα (for review of IL-6 action and metabolism see [30]). Stimulation of this receptor complex activates the *Janus kinase* (Jak)-STAT (signal transducer and activator of transcription) pathway which is known to induce *suppressor of cytokine signalling* (SOCS)-3 expression [31]. SOCS3 has been demonstrated to induce a serine phosphorylation of *insulin receptor substrate* (IRS)-1 which is known to impair insulin signalling [32]. These findings suggest a molecular mechanism by which IL-6 induces insulin resistance. Interestingly, it also has been found that, both, IL-6 serum levels and insulin resistance increase with age, indicating that IL-6 might also be involved in the molecular regulation of aging [33].

Several studies have suggested, that IL-6 might be important in the development of insulin resistance and type 2 diabetes, however, a causal relationship in humans has not been proven so far. Therefore, in the present study we have examined effects of Tocilizumab therapy on metabolic parameters. Tocilizumab is a human monoclonal antibody selectively inhibiting IL-6 signalling by blocking its receptor. Treatment with this antibody resulted in a significant reduction of insulin resistance as shown by a decrease of the HOMA-IR. We did not perform clamp experiments in the present study, however, instead we calculated the leptin to adiponectin ratio (LAR). This parameter has recently been shown to correlate very closely with the grade of insulin resistance measured by euglycemic clamp studies and HOMA index in more than 1000 human subjects [20]. In agreement with the decrease in HOMA-IR, we observed a significant reduction of the LAR during Tocilizumab treatment, indicating that insulin sensitivity is enhanced due to inhibition of IL-6 signalling in humans with rheumatoid disease. It also should be mentioned that in a recent report low adiponectin levels were found to be strongly associated with an increased risk for type 2 diabetes in humans [34]. The significant increase in adiponectin levels in our study are thus in keeping with favourable metabolic effects of Tocilizumab. One might argue that these effects on insulin resistance may be an indirect consequence of a reduction in overall inflammatory activity. However, in a clinical study with patients suffering from rheumatoid arthritis, the monoclonal anti-TNF-α antibody adalimumab reduced CRP levels without significant alteration of insulin resistance [14], indicating that changes in overall inflammatory activity per se do not necessarily alter insulin sensitivity. In agreement to these findings, no significant correlation was found between changes of CRP levels over time with changes of insulin levels, HOMA-IR as well as LAR in the present study (table 3).

**Table 3:** Correlation between changes of CRP levels with changes of markers of insulin resistance during the study period.

	*p*	P
LAR	0.049	0.909
HOMA-IR	−0.117	0.748
Insulin (µIU/ml)	−0.129	0.723

Spearman Correlation analysis, *p* =  correlation coefficient, P =  significances level.

Several studies have shown increased LDL-cholesterol and triglyceride levels in human subjects when treated with Tocilizumab [21], [35], [36]. This finding was also observed in our cohort, although the changes did not reach statistical significance. In addition, we also measured Lp (a) levels in response to IL-6 inhibition. High Lp (a) levels haven been found to be associated with an increased cardiovascular risk in human subjects in a meta-analysis including several prospective studies with a total number of 5436 human individuals and a mean follow up-period of 10 years [37]. Interestingly, in this meta-analysis the risk ratio did not change following adjustment for other cardiovascular risk factors suggesting that Lp (a) is an independent risk factor [37]. Lp (a) contains an unique glycoprotein termed apolipoprotein (apo)-a that is covalently linked to apoB100, the main apolipoprotein of LDL cholesterol. Apo (a) bears a striking homology to the sequence of plasminogen [38]. Lp (a) can inhibit fibrin clot lysis, most likely due to inhibition of tissue plasminogen activator (tPA)-mediated activation of plasminogen to its active form plasmin [39]. From a pathophysiological point of view, Lp (a) has been found in atherosclerotic lesions [40] where it is thought to link atherosclerosis to local thrombosis. Lp (a) is only present in humans and Old World monkeys and absent in common laboratory animals [41]. In humans, the serum concentration of Lp (a) is known to be mostly determined by genetic factors, however, secondary factors such as *acute phase response* (APR) have been shown to increase Lp (a) levels [42]. Moreover, it has been shown that IL-6 is an important pro-inflammatory cytokine in APR and a correlation between IL-6 and Lp (a) levels has been demonstrated [42]. The data obtained in the present study indicate that serum IL-6 levels are not only correlated to Lp (a) but that this cytokine is of causal relevance in the regulation of Lp (a) metabolism in humans. Since patients with inflammatory diseases are on increased cardiovascular risk compared to human subjects without immunological abnormalities [43], lowering of Lp (a) levels due to Tocilizumab treatment might be beneficial in such patients.

In the present study we examined the effect of IL-6 inhibition on insulin resistance in human subjects with a rheumatoid disease. Our data suggest, collectively, that IL-6 might be of causal relevance in impaired insulin sensitivity in humans. Furthermore, we show for the first time that inhibition of IL-6 signalling lowers Lp (a) levels which might positively affect the cardiovascular risk of human subjects. It also should be mentioned that the present study has some limitations which impact on the interpretation of the results. First, the number of subjects is very small and all the patients included in the study suffered from an immunological disease. Therefore it can not be excluded, that the improvement in insulin sensitivity is due to the reduction of overall inflammatory activity. Second, all of the patients were treated with Tocilizumab because of their inflammatory symptoms and no placebo control group was included, giving the study a clear observational character. Third, we did not perform hyperinsulinemic-euglycemic clamp experiments which are still considered to be the gold standard to assess insulin resistance. Fourth, the different drugs that the subjects had taken in addition to Tocilizumab provide an important interference in the study. Finally, the age of the 5 lean and 6 overweight/obese subjects range from 21 to 68 years and it is well known that age, menopausal status and the time of menstrual cycle affect IL-6 levels. However, the data obtained are in line with the results of Ogata et al. reported very recently [44], showing a significant improve in haemoglobin A1c levels of type 2 diabetic subjects with rheumatoid arthritis when treated with Tocilizumab also indicating favourable effects of Tocilizumab on glucose metabolism in humans.

From a pathophysiological point of view, the results obtained by Ogata et al. [44] and the data presented here give promising evidence for future studies using Tocilizumab as a tool to examine the effect of IL-6 on insulin sensitivity in human individuals not suffering from an immunological disease to further clarify the role of IL-6 in the pathogenesis of type 2 diabetes. Obviously, such studies are needed before any more definite conclusions can be drawn. However, from a clinical point of view, the data presented here might have direct implications for rheumatologists, since many patients with rheumatoid diseases additionally suffer from Lp (a) abnormalities and therefore might benefit immunologically and metabolically form a treatment with the IL-6 receptor antibody Tocilizumab.
